# Supplementary material for: Changes in Internal Structure and Dynamics upon Binding Stabilise the Nematode Anticoagulant NAPc2
Source: Biomolecules. 2024 Mar 30;14(4):421. doi: 10.3390/biom14040421 (PMC11048057; doi:10.3390/biom14040421)
Supplement: Supplementary file 1 [file biomolecules-14-00421-s001.zip › biomolecules-2940898-supplementary.pdf]

# Supporting Information

# Changes in internal structure and dynamics upon binding stabilise the nematode anticoagulant NAPc2.

Elaine Woodward[1] and Brendan M. Duggan[1,2]

1 Department of Biochemistry and Molecular Biology, Medical University of South Carolina, Charleston, SC 29425, USA.
2 Current address: Skaggs School of Pharmacy and Pharmaceutical Sciences, University of California, San Diego, La Jolla CA 92093. Correspondence: bmduggan@ucsd.edu

## Table of Contents

Figure S1. Secondary structure during the simulations....2
Figure S2. Comparison of NAPc2 experimental NMR and simulation-derived parameters....3
Figure S3. Active site gating residues and sodium ion in the fXa simulation....4
Figure S4. Ramachandran plots of NAPc2 residues E71-T82 in the NMR ensemble of NAPc2 and the crystal structure of the NAPc2-fXa complex....5
Figure S5. Correlation analysis of fXa in the free and bound simulations....6
Figure S6. Multiple sequence alignment of NAPs and other members of the trypsin inhibitor-like cysteine-rich family....7
Figure S7. NMR spectra of NAPs....8

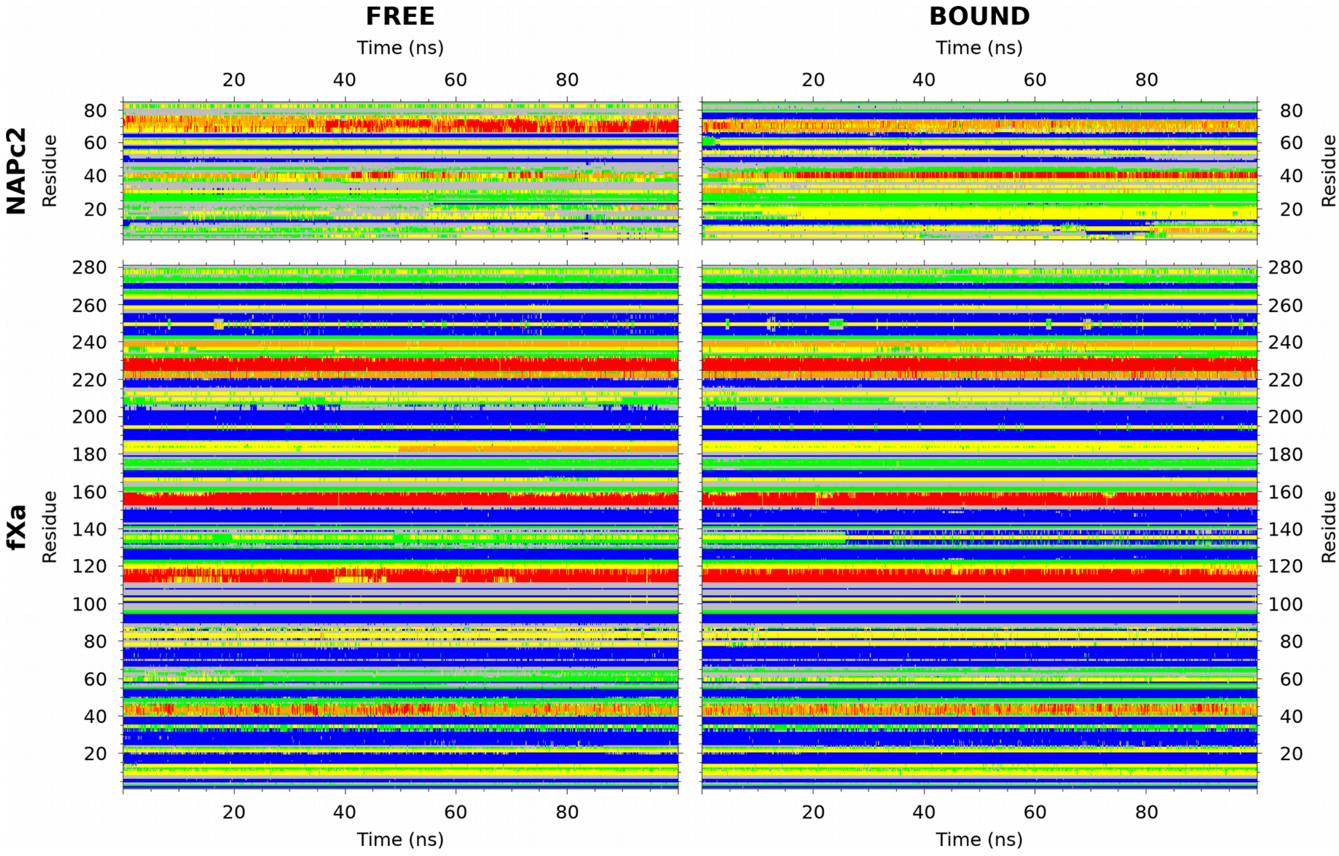


# Figure S1. Secondary structure during the simulations.

The secondary structure of NAPc2 and fXa was defined using the DSSP method and is plotted against simulation time. No secondary structure is shown as gray, β-strands as blue, $3_{10}$-helix as orange, α-helix as red, π-helix as brown, turns as yellow and bends as green. The top left panel shows the free NAPc2 simulation, the bottom left panel the free fXa simulation, the top right panel bound NAPc2 from the NAPc2-fXa simulation, and the bottom right panel shows bound fXa from the NAPc2-fXa simulation. Note that the extra β-strand formed by bound NAPc2 residues M75-I78 where it attaches to fXa is present throughout the simulation.

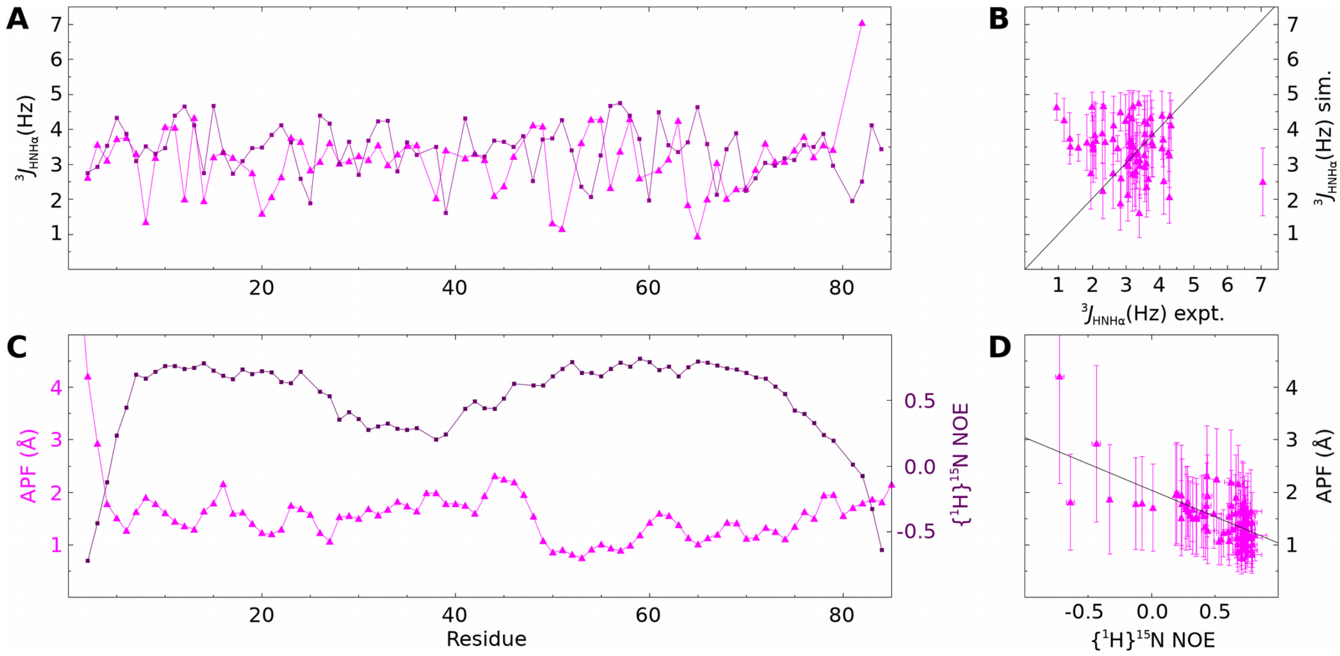


# Figure S2. Comparison of NAPc2 experimental NMR and simulation-derived parameters.

(A) $^3J_{HN,H\alpha}$ coupling constants calculated using the Karplus equation and φ angles in the free NAPc2 simulation (magenta triangles) and NMR measured values (purple squares) plotted against NAPc2 residue number. (B) Comparison of the experimental and simulation derived $^3J_{HN,H\alpha}$ coupling constants. Experimental uncertainties are smaller than the point markers. Theoretical uncertainties are ± one standard deviation. The black diagonal line is a line of best fit with $R^2$=0.86. The Pearson correlation coefficient is -0.22. The root-mean-squared deviation between the experimental and simulated values is 2.65, with the maximum deviation being 9.09 for T82 and the minimum deviation 0.005 for G16. (C) Atomic positional fluctuations (APF) for the backbone nitrogen atoms in the free NAPc2 simulation (magenta triangles) and {$^1$H}$^{15}$N heteronuclear NOE (purple squares) plotted against NAPc2 residue number. Increased APF and decreased heteronuclear NOE correspond to greater motion. (D) Comparison of the experimental {$^1$H}$^{15}$N heteronuclear NOE and simulation-derived APF. APF uncertainties are ± one standard deviation of the distance to the average structure. The black diagonal line is the line of best fit with $R^2$=0.44. The Pearson correlation coefficient is -0.66.

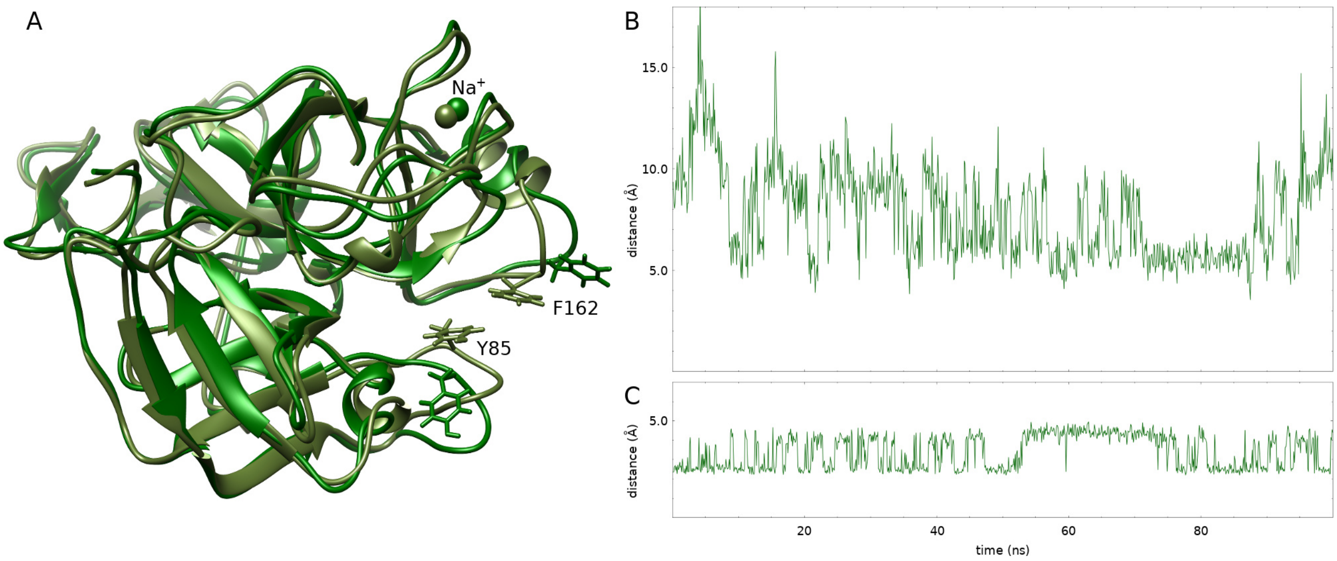


# Figure S3. Active site gating residues and sodium ion in the fXa simulation.

(A) The fXa structures at 4.2 ns (dark-green) and 93.6 ns (olive) are shown in ribbon format. The structures were superimposed on the backbone heavy atoms. The sidechains of the active site gating residues Y85 and F162 are shown as sticks and the bound sodium ion is shown as a sphere. (B) Distance between the Cζ atoms of the fXa active site gating residues Y85 and F162 over the course of the simulation. During the simulation the aromatic residues at the opening to the fXa active site approach each other then move apart, as reported in other simulations of fXa. Using a distance cutoff of 7.0 Å the trajectory can be separated into open and closed states, with the open state present for 53.6% of the trajectory and the closed state for the remaining 46.4%. Computing energies for the two states in the absence of solvent we find no significant difference between the open and closed states (open energy=-3071±146 kcal/mol, closed energy=-3116±159 kcal/mol). (C) Distance between the sodium ion and the R211 carbonyl oxygen over the course of the simulation.  The R211 carbonyl oxygen is the closest sodium-coordinating atom in the crystal structure of the NAPc2-fXa complex [Murakami et al 2007]. The distance jumps between two main values spending nearly equal time in each (2.6±0.2 Å, 49.7% and 4.2±0.3 Å, 50.3%), suggesting an equilibrium between two versions of the sodium co-ordinating site.

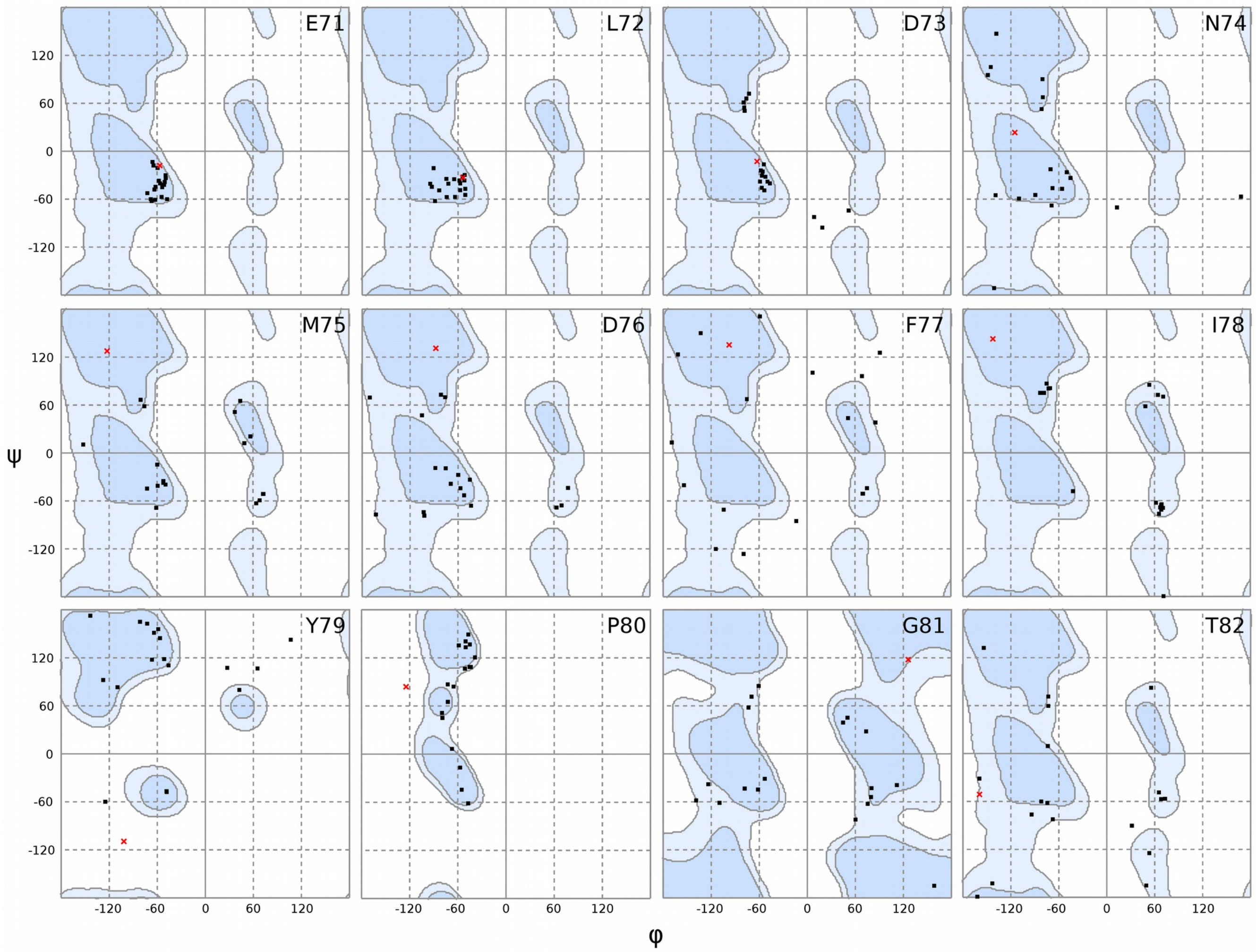


# Figure S4. Ramachandran plots of NAPc2 residues E71-T82 in the NMR ensemble of NAPc2 and the crystal structure of the NAPc2-fXa complex.

Allowed φ-ψ co-ordinates are shown in light blue with favoured co-ordinates a darker shade of blue. Black points represent the φ-ψ values from the 18 structures in the NMR ensemble of NAPc2 [Duggan et al 1999]. Red points represent values from the crystal structure of the NAPc2-fXa complex [Murakami et al 2007]. Ramachandran plots were generated with the Rampage server (http://raven.bioc.cam.ac.uk/rampage.php). Note how the values for E71, L72 and D73, where the NMR ensemble and crystal structure show a well defined α-helix, cluster in the α-helical region. In the crystal structure of the complex, residues M75-I78 form a β-strand, as indicated by the red points in the β-strand region. The values from the same residues in the NMR ensemble are scattered and do not cluster in the β-strand region, showing that the NMR ensemble does not include the bound conformation.

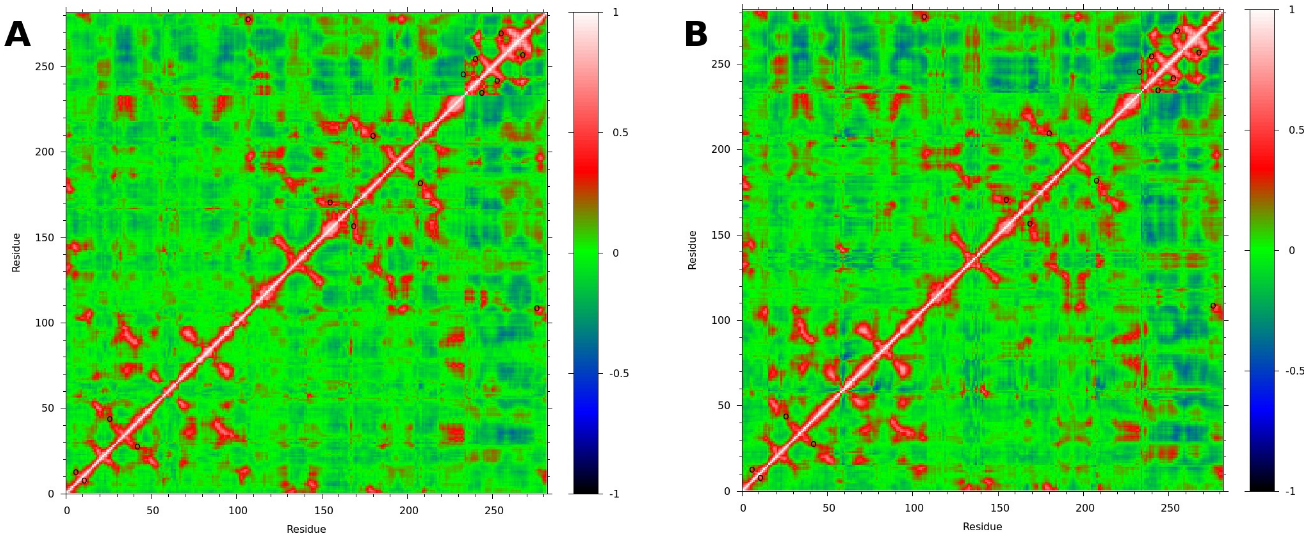


# Figure S5. Correlation analysis of fXa in the free and bound simulations.

Dynamic cross-correlation matrices were calculated using Cα atoms and the method[Hünenberger et al 1995] implemented in cpptraj[Roe et al 2013]. A correlation of 1 (red) indicates correlated motions, i.e. the atoms move in the same direction. A correlation of -1 (blue) indicates anti-correlated motions, i.e. the atoms move in opposite directions. A correlation of 0 (green) indicates no correlated motions. Open circles indicate disulfides. (A) Correlation matrix of fXa in the fXa simulation. (B) Correlation matrix of fXa in the NAPc2-fXa simulation. Residues 1-233 comprise the serine protease domain of fXa, also known as the heavy chain. Residues 234-281 comprise the EGF domain, also known as the light chain. The serine protease and EGF domains are linked by a disulfide bridge between residues 108 and 277. The only regions of correlated motions are residues close to the disulfides. Most of fXa moves independently.

```
AMCI-1        --------------EECG-PNEVFNTCGS--ACAPTCAQPKT---------RIC-TMQCR--    33
NAP5          -----------KAYPECG-ENEWLDDCGTQKPCEAKCNEEPPEEED-----PICRSRGCLLP    44
NAP6          -----------KAYPECG-ENEWLDVCGTKKPCEAKCSEE--EEED-----PICRSFSCPGP    42
NAP12         ------------AEKKCG-PNEWYDKCGTLKACEDRCNEDENEERDE----EACLSRACPGP    44
NAP9          MLLLVTQCSTNIVKPNCG-ENEEYDVCGN-RTCDLKCQYDGAEKKDEER-NAECLVRVCYD-    57
NAPc4         -------------KPSCG-ENQRYDECNR-KECDPKCKYDGTEEKDDEKP-VECLIRVCH--    44
NAP10         MLLLVSQCNA---NPSCG-ENERHDECSR-KECDPKCKYDGTEEKDDEKP-VVCLTRVCY--    54
NAP11         -------------NPSCG-ENERYDDCNR-KECDPKCKYDGTEEKDDEKP-VECLIRVCH--    44
NAPc3         -----------KATRKCG-ENEMYDPCGR-KECDQKCKYDGVEEEDDEEPNVQCLVRVCY--    47
NAPc2         -----------KATMQCG-ENEKYDSCGS-KECDKKCKYDGVEEEDDEEPNVPCLVRVCH--    47
NAP7          -----------KATRQCG-ENERYDSCGS-KECDKKCKYDGVEEEDDEEPNVPCLVRVCH--    47
NAP8          -----------KATMQCG-ENERYGSCGS-KECDKKCKYDGVEEEDDEEPNVPCLVRVCH--    47
ATI            -----------EAEKCTKPNEQWTKCGG---CEGTCAQKI----------VPCT-RECKPP    36
CE-1          ------------GQESCG-PNEVWTECTG---CEMKCGPDE---------NTPCP-LMCRRP    36
                              C: .N:    C.    C  .C                C  . C

AMCI-1        IGCQCQ--EGFLRNG-EGACVLPENC--------------    56
NAP5          PACVCK--DGFYRDTVIGDCVREEECDQHEIIHV------    77
NAP6          AACVCE--DGFYRDTVIGDCVKEEECDQHEIIHV------    75
NAP12         GVCMCD--PGFYRNK-NGKCVSKDDCEYDNMEFITFAP--    80
NAP9          GDCVCR--KGFYRNN-NGRCVTAEDCELDNMEFIYPKRK-    94
NAPc4         GDCVCK--DGFLRNN-NGACVKAGDCELDNMEFIYPNRK-    80
NAP10         GDCICR--DGFLRNK-NGACVKAEDCELDNMEFIYPNRK-    90
NAP11         GDCICR--DGFLRNK-NGACVKAEDCELDNMEFIYPNRK-    80
NAPc3         GDCVCE--EGYFRNK-NDICVKAEDCELDNMEFIYPGTQH    84
NAPc2         QDCVCE--EGFYRNK-DDKCVSAEDCELDNMDFIYPGTRN    84
NAP7          HDCVCQ--EGFYRNK-DDKCVSAENCELDNMEFIYPGTQH    84
NAP8          HDCVCQ--EGFYRNK-DDKCVSAENCELDNMEFIYPGTQH    84
ATI           -RCECIASAGFVRDA-QGNCIKFEDCPK------------    62
CE-1          -SCECSPGRGMRRTN-DGKCIPASQCPEH-----------    63
                C C    G: R.    . C:  . C
```

# Figure S6. Multiple sequence alignment of NAPs and other members of the trypsin inhibitor-like cysteine-rich family.

All eleven NAP sequences shown are from *Ancylostoma caninum*. AMCI-1 is a chymotrypsin inhibitor from *Apis mellifera*, ATI is a trypsin inhibitor from *Ascaris suum*, and CE-1 is a chymotrypsin and elastase inhibitor from *Ascaris suum*. Sequences were obtained from the NCBI. Residues highlighted in red are identical to NAPc2 residues. Residues identical in all sequences are listed below the alignment. Positions conserved in 12 or 13 of the 14 sequences are marked with ":". Positions conserved in 10 or 11 of the 14 sequences are marked with ".". For the sequences where the disulfide pairing has been determined it was found to be C1-C7, C2-C6, C3-C5, C4-C10 and C8-C9. The residues between cysteines 5 and 6 are cleaved by the target serine proteases and determine the specificity of the inhibitor. NAP5, NAP6 and NAP12 have been shown to inhibit fXa, while NAPc2, NAPc3 and NAPc4 inhibit fVIIa. The internal salt bridge that stabilises the bound conformation of NAPc2 is formed by E10, which is conserved in 13 of 14 sequences, and R58, which is conserved in all of the sequences.

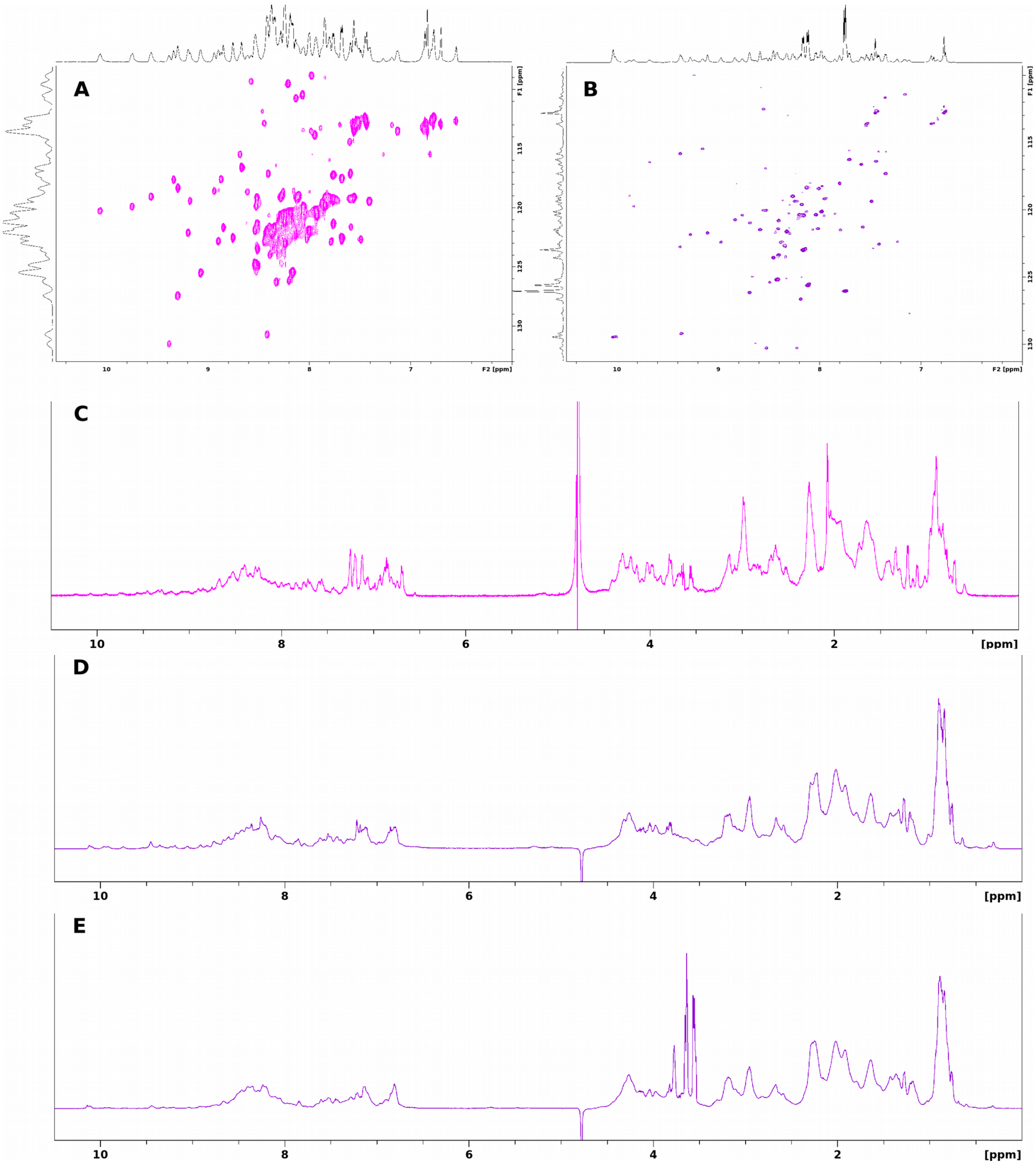


# Figure S7. NMR spectra of NAPs.

NMR spectra collected at 600 MHz and 298 K in 95% $H_2O$/5% $D_2O$. (A) 2D $^{15}N$ HSQC spectrum of NAPc2, consistent with spectra in previous work [Duggan et al 1999]. (B) 2D $^{15}N$ HSQC spectrum of NAP5. This spectrum was collected with higher resolution in the $^{15}N$ dimension than the spectrum in A so the peaks appear smaller. (C) 1D $^{1}H$ excitation sculpting spectrum of NAPc2. (D) 1D $^{1}H$ excitation sculpting spectrum of NAP5. (E) 1D $^{1}H$ excitation sculpting spectrum of NAP5 E10Q. The large peaks

at 3.77, 3.64 and 3.55 pm are due to contaminating glycerol. The dispersion of the peaks in all the spectra indicates that the proteins are folded. The similar profiles of the NAP5 and NAP5 E10Q $^{1}H$ spectra indicate that the mutation has not disrupted the structure.
